# In vitro hemodynamic performance of a blood pump for self-powered venous assist in univentricular hearts

**DOI:** 10.1038/s41598-024-57269-7

**Published:** 2024-03-23

**Authors:** Reza Rasooli, Henrik Holmstrom, Knut Erik Teigen Giljarhus, Ingunn Westvik Jolma, Jan Ludvig Vinningland, Charlotte de Lange, Henrik Brun, Aksel Hiorth

**Affiliations:** 1 Department of Energy Resources, Faculty of Science and Technology, University of Stavanger, 4036 Stavanger, Norway; 2 Department of Pediatric Cardiology, Division of Pediatric and Adolescent Medicine, Oslo University Hospital, Oslo, Norway; 3 Institute of Clinical Medicine, Faculty of Medicine, University of Oslo, Oslo, Norway; 4 Department of Mechanical and Structural Engineering and Materials Science, University of Stavanger, 4036 Stavanger, Norway; 5 Department of Chemistry, Bioscience and Environmental Engineering, University of Stavanger, 4036 Stavanger, Norway; 6 Norwegian Research Center (NORCE), Oslo, Norway; 7 Department of Pediatric Radiology, Sahlgrenska University Hospital, Gothenburg, Sweden; 8 Institute of Clinical Science, Sahlgrenska Academy, University of Gothenburg, Gothenburg, Sweden; 9 Section for Medical Cybernetics and Image Processing, The Intervention Centre, Oslo University Hospital Rikshospitalet, Oslo, Norway

**Keywords:** Fluid dynamics, Congenital heart defects, Biomedical engineering, Paediatric research

## Abstract

Univentricular heart anomalies represent a group of severe congenital heart defects necessitating early surgical intervention in infancy. The Fontan procedure, the final stage of single-ventricle palliation, establishes a serial connection between systemic and pulmonary circulation by channeling venous return to the lungs. The absence of the subpulmonary ventricle in this peculiar circulation progressively eventuates in failure, primarily due to chronic elevation in inferior vena cava (IVC) pressure. This study experimentally validates the effectiveness of an intracorporeally-powered venous ejector pump (VEP) in reducing IVC pressure in Fontan patients. The VEP exploits a fraction of aortic flow to create a jet-venturi effect for the IVC, negating the external power requirement and driveline infections. An invitro Fontan mock-up circulation loop is developed and the impact of VEP design parameters and physiological conditions is assessed using both idealized and patient-specific total cavopulmonary connection (TCPC) phantoms. The VEP performance in reducing IVC pressure exhibited an inverse relationship with the cardiac output and extra-cardiac conduit (ECC) size and a proportional relationship with the transpulmonary pressure gradient (TPG) and mean arterial pressure (MAP). The ideal VEP with fail-safe features provided an IVC pressure drop of 1.82 ± 0.49, 2.45 ± 0.54, and 3.12 ± 0.43 mm Hg for TPG values of 6, 8, and 10 mm Hg, respectively, averaged over all ECC sizes and cardiac outputs. Furthermore, the arterial oxygen saturation was consistently maintained above 85% during full-assist mode. These results emphasize the potential utility of the VEP to mitigate elevated venous pressure in Fontan patients.

## Introduction

Approximately 1% of neonates worldwide are born with a structural anomaly in their heart and great vessels^1,2^, known as congenital heart defects (CHD). Single-ventricle heart malformations, in which the patient only has one functional ventricle, are among the most fatal types of CHDs requiring immediate vascular reconstruction for survival. The palliative repair of these patient groups is typically achieved through a series of staged surgical interventions aimed at using the native ventricle for systemic circulation. The Fontan procedure, which was first carried out to treat tricuspid atresia^3,4^, has become the final stage of SV surgical palliation that puts the systemic and pulmonary circulations in series by creating a total cavopulmonary connection (TCPC) and diverting the venous return directly to the lungs. This peculiar circulation is based on elevated systemic venous pressure (SVP) exceeding pulmonary venous pressure (known as the Fontan paradox) and has almost inevitable adverse consequences^5^. Despite the success in early survival, the 30-year survival rate is only 43% to 70%^6–8^. The underlying pathophysiologic root cause of Fontan's high mortality and morbidity^9–11^ is widely acknowledged to be the chronic elevation in central venous pressure^12,13^ due to the lack of a subpulmonic ventricle, leading to systemic venous and lymphatic congestion, and eventually failure.

Notwithstanding decades of extensive research efforts, very limited progress has been made toward reversing the Fontan paradox^14^, and cardiac transplantation has remained the sole definitive therapy. As the population of late survivors with Fontan circulation continues to grow and donor organ shortages persist, the management of Fontan failure has become a significant clinical challenge, and innovative strategies are urgently required^15^. Attempts aimed at addressing the Fontan paradox have primarily focused on optimizing the TCPC anatomy to improve blood energetics through novel surgical pathways, such as Y-grafts^16–21^. These modifications, however, fail to address the physiologic deficit of a ventricle as the right-side driving pump and thus have little impact on the Fontan outcomes^22^. Resolving the Fontan paradox and its associated diseases requires addressing the lack of a subpulmonary power source to reinstate the biventricular-like hemodynamics. One alternate approach involves the use of existing standard mechanical circulatory support (MCS) devices to provide cavopulmonary or systemic assist^23–26^. MCS devices, on the other hand, are often employed to provide systemic support for end-stage Fontan failure as a bridge-to-transplantation therapy, and their application for cavopulmonary assist is limited and linked with poor outcomes due to technological mismatches^25–28^. This is primarily due to the fact that cavopulmonary assist requires a device capable of providing high flows with low pressure rise, which is a distinct requirement from existing MCS systems that typically operate at higher differential pressures^29^.

The design and development of a Fontan-specific cavopulmonary assist device (CPAD) continues to be a complex research challenge with limited solutions proposed over the past few decades^30–35^. The majority of the proposed CPADs are not fully implantable due to their reliance on an ex vivo power source, which can lead to a heightened risk of driveline infections and a diminished quality of life. Therefore, the development of an effective and fully implantable CPAD that can provide reliable and sustained support to the Fontan circulation remains an essential goal of current research efforts. More importantly, the suggested strategies are not capable of improving the pulsatility of non-pulsatile pulmonary blood flow, which is considered a major contributor to systemic venous hypertension. Studies conducted on both animal models and human subjects have highlighted the adverse consequences of non-pulsatile pulmonary blood flow, including the development of pulmonary vascular changes, endothelial dysfunction, reduced nitric oxide synthetase, and increased pulmonary vascular resistance^36–38^. These findings emphasize the pivotal role of pulmonary flow pulsatility in the pathogenesis of postoperative complications in Fontan patients. Therefore, novel approaches that address this critical hemodynamic factor are necessary to mitigate the long-term risks associated with Fontan physiology.

Numerous studies have proposed various Fontan venous assist strategies to address the limitations associated with ex vivo power sources and driveline infections. One such approach, proposed by Pekkan et al.^39^, involves an integrated turbine-pump system that harnesses a portion of aortic flow to drive a turbine coupled to a pump. While in vitro experiments have demonstrated the ability of the system to provide proper venous support, the presence of turbine-to-pump leakage poses a significant challenge, affecting the system's overall performance. Computational simulations have suggested that an alternative approach, such as a shunt that injects a portion of aortic flow into either the pulmonary arteries^40^ or inferior vena cava (IVC)^41^, can reduce IVC pressure. However, unsupported shunts are highly susceptible to fluctuations due to the pulsatile nature of the aortic flow, which may significantly affect the system's clinical feasibility and performance. Another recently proposed approach involves the use of a conduit that connects the ascending aorta to the superior vena cava (SVC) and pulmonary arteries anastomosis^42^. Nevertheless, computational simulations have revealed an IVC pressure drop of less than 1 mm Hg and uneven pulmonary flow distribution. Our group earlier proposed a passive and fully implantable solution for elevated IVC pressure with a theoretically favorable impact on the Fontan local hemodynamics^43^. The proposed solution features a structurally simple design, clinical feasibility, and an intracorporeal power source while avoiding rotating components. The present study aims to provide thorough in vitro validation for the proposed solution and to investigate its impact on the Fontan global hemodynamics using a pulsatile in vitro mock-up circulatory loop emulating single-ventricle Fontan circulation. The in vitro experiments conducted in this study simulate physiological conditions corresponding to the pediatric population, specifically targeting individuals with varying degrees of pulmonary hypertension characterized by preserved ejection fraction and ventricular function.

## Methods

### Institutional review board and Informed consent statements

The study was conducted according to the guidelines of the Declaration of Helsinki. It is part of a project, approved by the Regional Committees for Medical Research Ethics South-East Norway (nr 148926, approved 2020-10-05) and the Data protection officer at Oslo University Hospital (nr 20/20961, approved 2021-02-16). The patient-specific model used in this study is based on anonymous data from Oslo University Hospital. Patient consent was waived in this case due to the use of completely anonymized data without the possibility to identify a patient.

### Proposed blood pump

**Figure 1:**
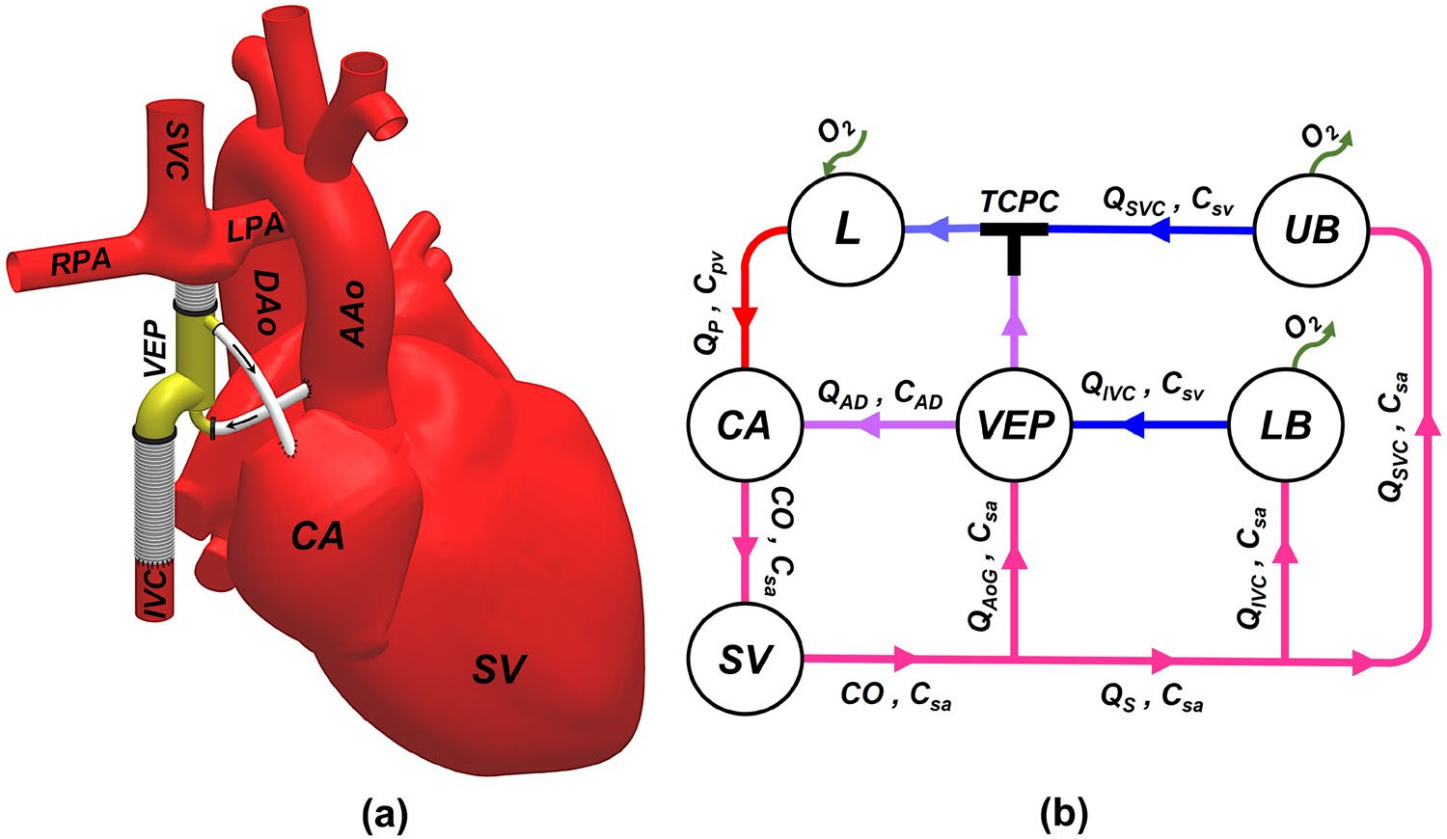
Anatomical positioning of the proposed venous ejector pump (**a**) and the circulation loop of the VEP-assisted Fontan (**b**) is schematically illustrated. *IVC* Inferior vena cava, *SVC* superior vena cava, *RPA* right pulmonary artery, *LPA* left pulmonary artery, *AAo* ascending aorta, *DAo* descending aorta, *UB* upper body, *LB* lower body, *L* lungs, *AoG* aortic graft, *AD* atrial discharge, *SV* single-ventricle, *CA* common atrium, *TCPC* total cavopulmonary connection, *CO* cardiac output, *Q* flow rate, *C* oxygen concentration, *sa* systemic arterial, *sv* systemic venous, *pv* pulmonary veins, *Q*_*P*_ pulmonary flow, *Q*_*S*_ systemic flow. The line colors on the right side schematically represent the blood oxygen saturation with blue and red being the systemic venous and pulmonary veins oxygen levels, respectively.

**Figure 2:**
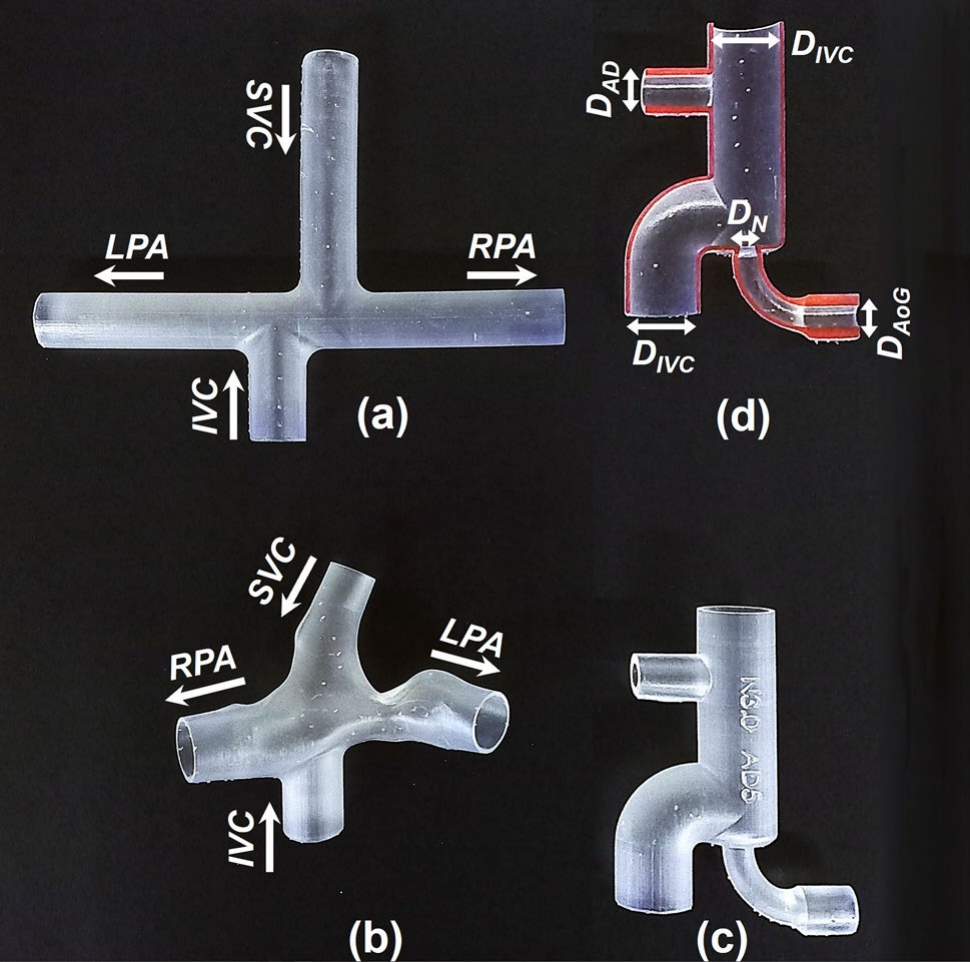
Stereolithographic TCPC phantoms and the VEP prototypes. (**a**) idealized TCPC with 13 mm ECC size, (**b**) 3D reconstructed patient-specific TCPC, (**c**) VEP prototype with aortic nozzle, atrial discharge, and ECC diameters of 3 mm, 5 mm, and 13 mm respectively, and (**d**) cross-section of the printed VEP prototype. *IVC* Inferior vena cava, *SVC* superior vena cava, *RPA* right pulmonary artery, *LPA* left pulmonary artery, *AoG* aortic graft, *AD* atrial discharge, *ECC* extra-cardiac conduit, *TCPC* total cavopulmonary connection, *VEP* venous ejector pump.

The venous ejector pump (VEP) operates on the principle of two interrelated mechanisms aimed at lowering IVC pressure. These mechanisms include the Venturi effect, generated by the high-momentum aortic graft jet, and the suction effect of the atrium discharge flow (Fig. 1). The VEP incorporates two inlets, one each for the collection of blood from the IVC and its connection to the aortic graft. The device also features two outlets, one each for the discharge of venous blood into the pulmonary arteries and the atrium, respectively, as depicted in Fig. 2c,d. The aortic graft, referred to as AoG, functions as a left-to-right shunt by being anastomosed to the ascending aorta, thereby allowing a portion of the aortic flow to be injected into the low-momentum IVC flow, which is subsequently connected to the extra-cardiac conduit (ECC) port of the VEP. Similarly, the atrial discharge (AD) mechanism, functioning as a right-to-left shunt analogous to a fenestration, helps to improve preload and provide additional IVC pressure drop. Three sizes of 5 mm, 6 mm, and 7 mm were considered for the atrial discharge diameter based on typical fenestration sizes used in Fontan patients^44^. Correspondingly, a 4 mm aortic graft inlet size was deemed congruent with established clinical practices pertaining to aortopulmonary shunts^45^. In accordance with prior studies^40,41,43^, a range of aortic nozzle sizes spanning from 2.0 mm to 3.5 mm was taken into consideration.

### Total cavopulmonary connection models

Planar double-inlet double-outlet intersecting junction configurations with circular cross-sections and one diameter inlets offset (horizontal distance between caval midpoints) representing idealized TCPCs (Fig. 2a) were designed in Autodesk inventor (Autodesk, California, USA). The inlets and outlets correspond to the vena cavae (IVC and SVC) and pulmonary arteries (RPA and LPA), respectively. The pulmonary arteries' diameter was considered to be 13 mm, corresponding to the typical size in the pediatric population^46^. The vena cavae and pulmonary arteries anastomoses were rounded with a radius of curvature of 5 mm to create a more efficient TCPC^47^. A patient-specific geometry using reconstructed 3D magnetic resonance (MR) images of a TCPC circulation in a 16-year-old patient^43^ with an ECC size of 16 mm, was also employed to simulate a more complex and realistic cavopulmonary connection (Fig. 2b).

### Prototype manufacturing

All VEP prototypes and TCPC phantoms were realized and manufactured by stereolithography 3D printing (Form 3+, Formlabs, MA, USA) using clear V4 photopolymer resins followed by a post-washing process (Form wash) to ensure the removal of resin residual and improve the surface roughness quality (Fig. 2). A UV-light post-curing was applied to all printed models for 30 min at a temperature of 40 °C to enhance rigidity and reduce water absorbability.

### Pulsatile in vitro mock circulatory loop

**Figure 3:**
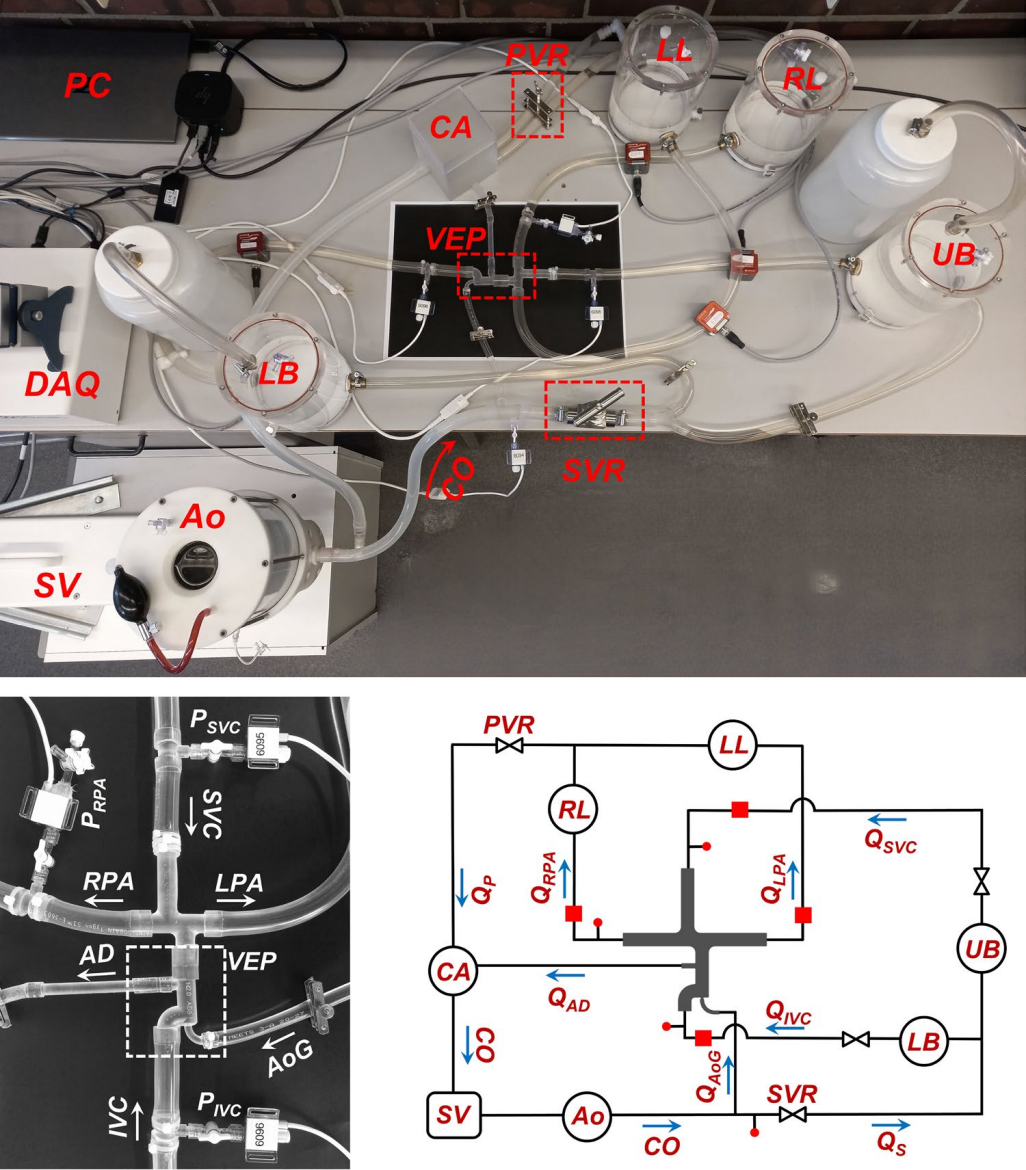
Pulsatile in vitro mock-up Fontan circulatory loop used in this study (top), close-up of the TCPC + VEP (bottom left), and the schematic representation of the flow loop (bottom right). The red dots, red squares, circles, and double triangles represent the pressure measurement points, flow meters, compliance chambers, and resistances, respectively. *IVC* Inferior vena cava, *SVC* superior vena cava, *RPA* right pulmonary artery, *LPA* left pulmonary artery, *SV* single-ventricle, *Ao* aorta, *CO* cardiac output, *AoG* aortic graft, *SVR* systemic vascular resistance, *Q*_*S*_ systemic flow, *LB* lower body, *UB* upper body, *LL* left lung, *RL* right lung, *PVR* pulmonary vascular resistance, *Q*_*P*_ pulmonary flow, *CA* common atrium, *AD* atrial discharge, *Q* flow rate, *P* pressure.

A reduced-order lumped parameter mock-up circulatory loop representing the Fontan single-ventricle circulation was developed in this study (Fig. 3) to assess the VEP performance, similar to earlier validated works^48,49^. The in vitro flow loop system comprises the left ventricle, aorta, upper and lower body circulation, TCPC, right and left pulmonary arteries, and common atrium to make up the systemic and pulmonary circulations. An advanced and highly versatile pulsatile pump (PD-1100, BDC Laboratories, Colorado, USA) was employed to simulate physiological SV-aorta hemodynamics and ventricular pumping action. The pump was coupled to a hemodynamic flow conditioner, which included directional valves and a trapped-air compliance chamber for fine-tuning the arterial pulse pressure. A high-resolution needle valve (Swagelok, Ohio, USA) representing the systemic vascular resistance (SVR) was utilized to precisely set the mean aortic pressure. Trapped-air compliance chambers were used to account for the capacitance of different vascular beds. A calibration procedure was performed for each chamber to obtain the compliance characteristic curves. To realistically represent the pediatric population, the compliance values considered for each vascular bed segment were taken from physiological data available in clinical reports and literature^49,50^. Briefly, compliance values of 3.12 mL/mm Hg, 3.85 mL/mm Hg, 2.14 mL/mm Hg, and 2.14 ml/mm Hg were considered for the upper body, lower body, right and left pulmonary arteries, respectively. The lower body compliance includes liver and IVC compliances as well. Tygon 3603 flexible tubing (Saint-Gobain, Courbevoie, France) was used for the connections. Pulmonary vascular resistance (PVR) was simulated using a clamp-on flow control pinch valve. Pressure measurements were performed at four different locations (aorta, IVC, SVC, and RPA) using non-invasive disposable pressure transducers (BDC Laboratories, Colorado, USA). The aortic pressure measurement point was located 10 mm cranially from the aortic graft anastomosis. Four ultrasonic clamp-on flow meters (Sonotec GmbH, Saale, Germany) were employed to measure the systemic (IVC, SVC) and pulmonary (RPA, LPA) flow rates. Aortic graft and atrial discharge flow rate values were calculated using the continuity equation and the measured systemic and pulmonary flows. Each flow meter was calibrated for the specific tubing and fluid prior to the experiments. The measurement uncertainties regarding the pressure transducers and flowmeters were approximately 2%. A non-Newtonian single-phase blood analog (a solution of xanthan gum in water with concentration of 0.04% by weight) that has been reported to closely match the blood viscosity of Fontan patients^51,52^ was prepared and used as the working fluid.

### Established hemodynamic conditions

The TCPC-state experiments representing the baseline Fontan circulation without the VEP were first established by clamping both aortic and atrial discharge grafts. Two cardiac outputs of 2.4 L/min and 3.5 L/min were considered for the TCPC-state experiments to resemble the postoperative pediatric Fontan conditions. The pulmonary arterial pressure was first set in TCPC-state circulation by tuning the PVR to simulate a hypertensive pulmonary vascular condition with relevant transpulmonary pressure gradient (TPG) values^53^. The IVC/SVC systemic flow distribution was approximately kept at a ratio of 60/40 in all experiments and device operation modes using the clamp-on flow controllers. A physiological aortic pressure waveform with a mean arterial pressure (MAP) and pulse pressure of 85 mm Hg and 40 mm Hg, respectively, was generated during the TCPC-state circulation. The heart rate was set to 70 bpm to represent the resting condition in Fontan patients^54^. The full-assist operation mode was simulated by instantaneously introducing the VEP into the TCPC-state circulation and unclamping the aortic and atrial discharge grafts. The VEP is prone to two failure scenarios: occlusion of aortic graft (AoG failure mode) or atrial discharge graft (AD failure mode). To evaluate the fail-safe feature of the VEP, each failure scenario was simulated by clamping the respective graft and recording any observed deviations in hemodynamic indices.

### Experimental procedure and parameters

Aside from VEP geometrical characteristics like aortic nozzle and atrial discharge diameters, the major parameters influencing performance were MAP, TPG, ECC size, and heart rate. In the initial phase, in vitro experiments were conducted to simulate both full assist and failure modes across a spectrum of aortic nozzle and atrial discharge sizes, as detailed in Section "Proposed blood pump". The ECC size was considered to be 13 mm, with MAP set at 85 mm Hg, TPG at 10 mm Hg, and heart rate at 70 bpm. This comprehensive dataset served as the foundation for identifying optimal sizes that met stringent criteria, namely:

Maintaining a cardiac output to systemic flow ratio below 1.5 (considered a physiological threshold for ventricular load and an estimation of stroke work during full assist over baseline Fontan).

Ensuring arterial oxygen saturation remained above 80% in both full assist and failure modes.

Subsequently, designs meeting these criteria were subjected to a detailed exploration of the impact of the aforementioned parameters. Specifically, the influence of MAP was scrutinized through three distinct values: 65 mm Hg, 85 mm Hg, and 105 mm Hg, while keeping other hemodynamic parameters consistent with conditions described in Section "Established hemodynamic conditions". Furthermore, to quantify the effects of ECC size and TPG, a range of diameters from 13 to 20 mm was considered to accommodate size variability in pediatric Fontan patients. For each ECC size, experiments were conducted with three TPG values—6 mm Hg, 8 mm Hg, and 10 mm Hg—corresponding to Pulmonary Vascular Resistance Index (PVRI) values of 2.14 WU.m2, 2.86 WU.m2, and 3.57 WU.m2, respectively. All other hemodynamic parameters were maintained at identical levels to those outlined in Section "Established hemodynamic conditions". Finally, the impact of heart rate variation was studied using three values: 50 bpm, 70 bpm, and 90 bpm, while keeping all other conditions consistent with those described in Section "Established hemodynamic conditions". This systematic exploration contributes to a comprehensive understanding of the interplay of parameters in VEP performance. The summary of in vitro experiments conducted in the present study is provided in Table 1.

**Table 1:** Summary of the in vitro experiments conducted in the present study.

EXP NO	(I)	(II)	(III)	(IV)	(V)
D_N_ (mm)	2.0, 2.5, 3.0, 3.5	2.0, 2.5	2.0	2.0	2.0
D_AD_ (mm)	5, 6, 7	5	5	5	5
D_ECC_ (mm)	13	13	13, 16, 18, 20	18	PS
CO_TCPC_ (L/min)	2.4, 3.5	2.4, 3.5	2.4, 3.5	2.4, 3.5	2.4, 3.6
P_Ao_ (mm Hg)	85	65, 85, 105	85	85	85
P_PA_ (mm Hg)	15	15	11, 13, 15	15	15
HR (bpm)	70	70	70	50, 70, 90	70

*EXP NO* experiment number, *D*_*N*_ aortic nozzle diameter, *D*_*AD*_ atrial discharge diameter, *D*_*ECC*_ extra-cardiac conduit diameter, *CO*_*TCPC*_ TCPC-state cardiac output, *P*_*Ao*_ mean aortic pressure, *P*_*PA*_ mean pulmonary artery pressure, *HR* heart rate, *PS* patient-specific.

### Arterial oxygen saturation tracking

The introduction of the VEP into the Fontan circulation would result in a slight arterial oxygen desaturation due to the presence of atrial discharge. Monitoring arterial oxygen levels is essential for ensuring sufficient oxygenation of organs and tissues. Considering Fig. 1.b, the oxygen equilibrium inside the atrium can be written as:

1 $${Q}_{P}{C}_{pv}+{Q}_{AD}{C}_{AD}=CO\cdot {C}_{sa}$$

where ${Q}_{P}$, ${C}_{pv}$, ${Q}_{AD}$, ${C}_{AD}$, $CO$, and ${C}_{sa}$ are pulmonary flow rate, pulmonary veins oxygen saturation, atrial discharge flow rate, atrial discharge oxygen saturation, cardiac output, and arterial oxygen saturation, respectively. The relation between atrial discharge and venous oxygen saturation can be defined as:

2 $${C}_{AD}={C}_{sv}+m\cdot ({C}_{sa}-{C}_{sv})$$

where ${C}_{sv}$ is the venous oxygen saturation and $m$ is a dimensionless coefficient indicating the level of mixing for aortic graft and IVC flows. A value of zero for “m” indicates that the aortic flow has no contribution to the atrial discharge. To realistically quantify the value of coefficient m and avoid overestimation, computational fluid dynamics (CFD) simulations incorporating steady-state Reynolds Averaged Navier Stokes and shear-stress transport (SST) k-ω turbulence model were conducted for each specific VEP + TCPC geometry. The cycle-averaged flow rate values obtained during the in vitro experiments were assigned as the mass flow inlet boundary conditions for IVC, SVC, and aortic graft inlet. Outlet boundary conditions using the experimental data were considered for RPA, LPA, and atrial discharge. A passive scalar transport equation coupled with the velocity field was also numerically solved to determine the aortic graft flow contribution to the atrial discharge. For this purpose, a value of 1 at the aortic graft inlet and zero elsewhere was considered for the passive scalar. The walls were assumed rigid and impermeable with zero flux. The surface averaged value of the passive scalar at the atrial discharge outlet was considered as the coefficient m. Please refer to^43^ for more details on the CFD settings. The relation between arterial and venous oxygen saturation in Fontan patients was defined as^55,56^:

3 $${C}_{sv}={0.6C}_{sa}$$

The pulmonary veins' oxygen saturation (${C}_{pv}$) was considered to be 95% to avoid overestimation.

## Results

### Impact of geometrical parameters and cardiac output

#### Full assist mode

The hemodynamic impact of different aortic nozzle (D_N_) and atrial discharge (D_AD_) sizes was investigated by incorporating each VEP prototype into the Fontan mock flow loop. The results in this section correspond to the EXP NO (I) in Table 1. The introduction of the VEP into the circulation resulted in a drop in arterial blood pressure (ABP) due to the presence of the aortic graft that draws a fraction of the cardiac output. To maintain hemodynamic homeostasis in the event of reduced blood pressure, the arterial baroreceptor reflex would regulate the ABP by increasing the SVR and CO, the two primary control mechanisms affecting ABP. This study assumes that the regulation of ABP is controlled by the CO since the interrelation between SVR and CO response is not quantitatively known. More importantly, this would reveal the least efficient performance of VEP full assist since an increase in SVR would result in a lower increase in CO, and consequently lower venous pressure rise and better performance. Please refer to Supplementary Material for more details on this assumption. For this purpose, the reduced ABP was adjusted back to its TCPC-state condition (both MAP and pulse pressure) by incrementally increasing the CO and monitoring the aortic pressure without changing the SVR. The CO adjustment was achieved by increasing the stroke volume and keeping the heart rate identical to its TCPC-state value (70 bpm).

**Figure 4:**
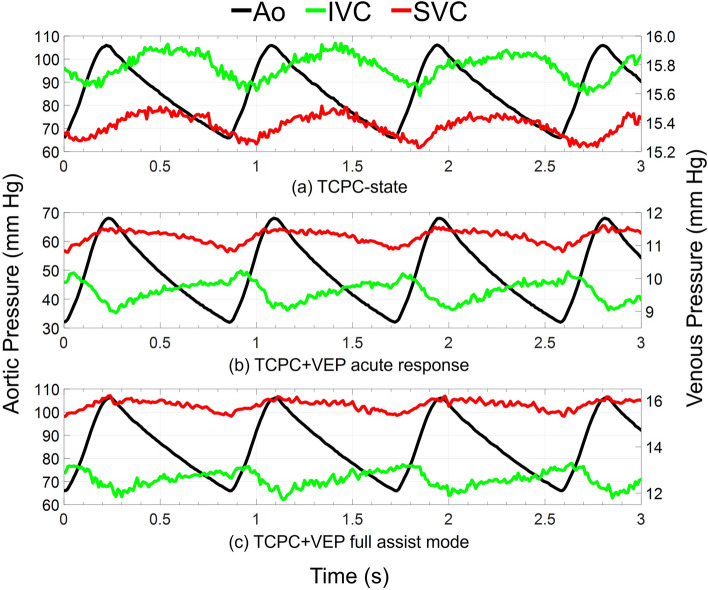
Obtained aortic and venous pressure waveforms during pulsatile in vitro experiments. TCPC-state represents the baseline Fontan hemodynamics without the VEP. TCPC + VEP acute response and full assist correspond to the hemodynamic conditions after the instantaneous introduction of the VEP into the baseline Fontan circulation without and with arterial pressure and cardiac output adjustments, respectively. The results correspond to the VEP with aortic nozzle, atrial discharge, and ECC sizes of 2.5 mm, 5.0 m, and 13 mm, respectively. The TCPC-state CO and MAP are 2.4 L/min and 85 mm Hg, respectively. *IVC* Inferior vena cava, *SVC* superior vena cava, *Ao* aorta, *TCPC* total cavopulmonary connection, *VEP* venous ejector pump, *ECC* extra-cardiac conduit, *CO* cardiac output, *MAP* mean aortic pressure.

**Figure 5:**
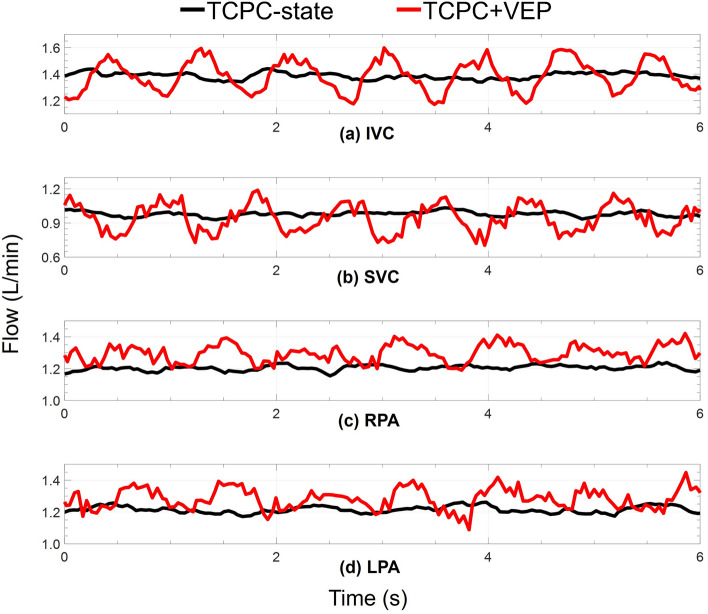
Systemic venous and pulmonary artery flow waveforms obtained during pulsatile in vitro experiments. TCPC-state represents the baseline Fontan hemodynamics without the VEP and TCPC + VEP corresponds to the full assist mode of the VEP. The results correspond to the VEP with aortic nozzle, atrial discharge, and ECC sizes of 2.5 mm, 5.0 m, and 13 mm, respectively. The TCPC-state CO and MAP are 2.4 L/min and 85 mm Hg, respectively. *IVC* inferior vena cava, *SVC* superior vena cava, *RPA* right pulmonary artery, *LPA* left pulmonary artery, *CO* cardiac output, *MAP* mean aortic pressure, *TCPC* total cavopulmonary connection, *VEP* venous ejector pump.

The instantaneous variation of pressure and flow waveforms for the VEP with a D_N_ of 2.5 mm and D_AD_ of 5 mm is provided in Figs. 4 and 5, respectively, for TCPC-state, TCPC + VEP acute, and TCPC + VEP full assist conditions with a TCPC-state CO of 2.4 L/min. TCPC + VEP acute represents the immediate hemodynamic response after VEP inclusion into the mock circulation without any hemodynamic adjustment. The activation of VEP remarkedly changed the venous pressure and flow waveforms with a significant improvement in the pulsatility levels. Clearly, the phase shift between aortic and venous pressure present at the TCPC-state almost vanishes during VEP-assisted circulation as the highly pulsatile AoG flow dominates. During the systolic phase, the pressure dynamics in the IVC and SVC exhibit complex interplay that reflects the underlying hemodynamic forces. As the aortic pressure increases during systole, a corresponding increase in AoG flow ensues as it is driven by the arterial-venous pressure gradient. Consequently, the SVC pressure rises due to the impingement of IVC and SVC flows, while the IVC pressure shows a slight initial rise followed by a sharp drop. The AoG flow and IVC pressure interplay is governed by two competing effects, both arising from the increased AoG flow. The first effect, driven by the increased pulmonary flow (PVR-effect), results in an increased IVC pressure due to the greater back pressure, as the PVR remains constant. The second effect is due to greater jet flow momentum transfer to the IVC section (Venturi-effect), leading to stronger Venturi effects and consequently lower pressure levels. Thus, the PVR-effect predominates during the initial phase of the systole, justifying the slight increase in the IVC pressure.

**Figure 6:**
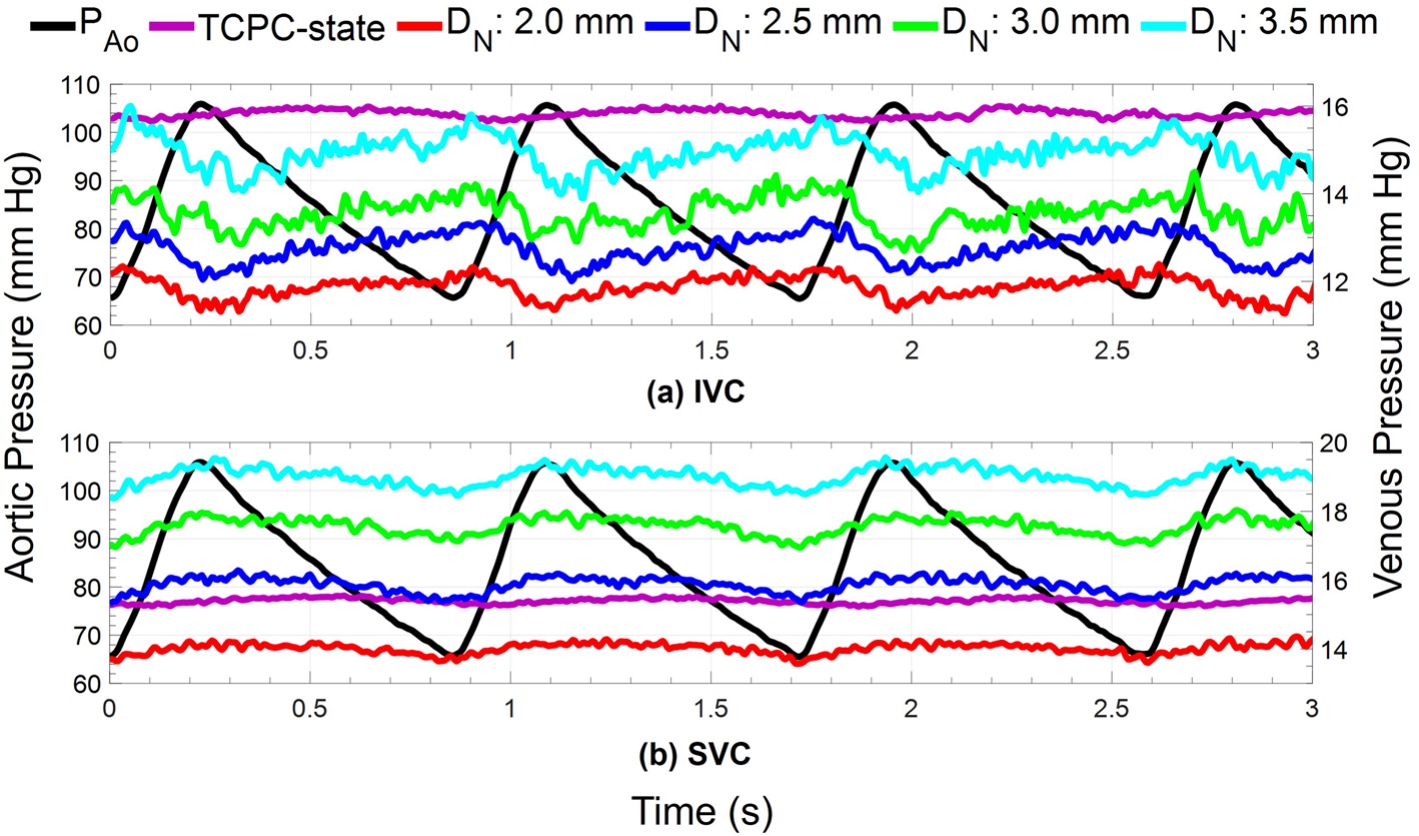
Venous pressure waveforms during both TCPC-state and the full assist operation mode of the VEP with different aortic nozzle sizes. TCPC-state represents the baseline Fontan hemodynamics without the VEP. The results correspond to the VEP with atrial discharge and ECC size of 5 mm and 13 mm, respectively. The TCPC-state CO and MAP are 2.4 L/min and 85 mm Hg, respectively. *IVC* inferior vena cava, *SVC* superior vena cava, *Ao* aorta, *D*_N_ aortic nozzle diameter, *TCPC* total cavopulmonary connection, *ECC* extra-cardiac conduit, *CO* cardiac output, *MAP* mean arterial pressure.

**Figure 7:**
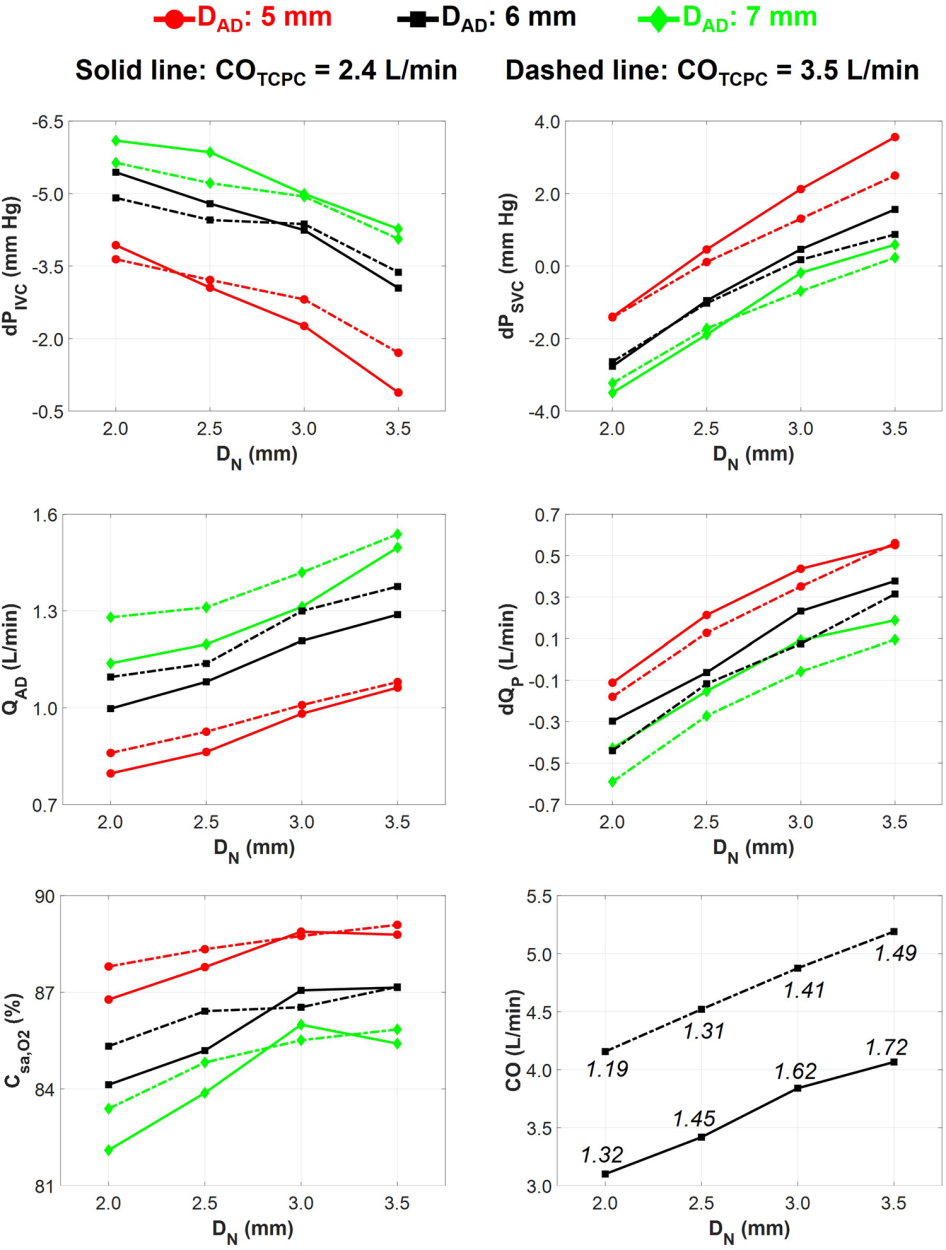
Impact of aortic nozzle size, atrial discharge size, and TCPC-state CO on the VEP-assisted Fontan hemodynamics during full assist operation mode. The ECC size and MAP in these experiments are 13 mm and 85 mm Hg, respectively. TCPC-state represents the baseline Fontan hemodynamics without the VEP. *IVC* inferior vena cava, *SVC* superior vena cava, *dP* pressure change as compared to TCPC-state, *Q*_*AD*_ atrial discharge flow rate, *dQ*_*P*_ pulmonary flow change as compared to TCPC-state, *C*_*sa, O2*_ arterial oxygen saturation, *CO* cardiac output, *D*_*N*_ aortic nozzle diameter, *D*_*AD*_ atrial discharge diameter. The numbers on the CO chart represent the ratio of CO over systemic flow during full assist which is an estimation of stroke work during full assist over the baseline Fontan.

The mean arterial pressure experienced a reduction of 32%, 42%, 48%, and 52% during VEP acute mode with aortic nozzle diameters of 2.0 mm, 2.5 mm, 3.0 mm, and 3.5 mm, respectively, with a TCPC-state CO of 2.4 L/min. The aortic pressure during VEP acute mode was higher (~ 10%) in the case of the TCPC-state CO of 3.5 L/min as compared to 2.4 L/min. These results correspond to the AD graft size of 5 mm; however, the values were almost identical for other diameters. The sample VEP-assisted venous pressure waveforms for various aortic nozzle sizes are provided in Fig. 6 for an AD graft size of 5 mm and TCPC-state CO of 2.4 L/min. The summary of key Fontan hemodynamic indices is illustrated in Fig. 7. The systemic flow rate during the full-assist was in the range of 2.3–2.4 L/min and 3.4–3.5 L/min for TCPC-state CO of 2.4 L/min and 3.5 L/min, respectively, almost identical to pre-VEP values. Larger aortic nozzle sizes resulted in higher venous pressure levels but with similar waveform patterns. As the nozzle grows larger, given that the AoG flow is purely driven by the arterial-venous pressure gradient, an increase in AoG flow ensues due to lower resistance. Obviously, the PVR-effect of an increased AoG flow predominates, leading to higher venous pressure levels. However, a significant increase in the isolated Venturi-effect was observed as the nozzle became larger by looking at the inferior cavopulmonary pressure head. More importantly, an average pulmonary pulse pressure of 1.06 mm Hg, 1.25 mm Hg, 1.50 mm Hg, and 1.63 mm Hg was observed during full assist with aortic nozzle diameters of 2.0 mm, 2.5 mm, 3.0 mm, and 3.5 mm, respectively, which is a significant improvement compared to TCPC-state pulmonary pulse pressure of 0.38 mm Hg. The values were averaged for different AD sizes and cardiac outputs as they exhibited minimal impact on pulmonary pulsatility. The AoG flow rate was found to be independent of AD size and TCPC-state CO, owing to virtually the same values of the arterial-venous pressure gradient. This also justifies the lower drop in MAP during acute response for higher TCPC-state CO, as identical AoG flow rate results in lower flow percentage drawn from the systemic circulation and consequently higher MAP levels. The impingement of IVC and SVC flows led to higher SVC pressure values for larger nozzle diameters. Nozzle diameters of 2.0 mm and 2.5 mm almost did not cause any SVC pressure elevation as compared to TCPC-state.

Expectedly, larger AD sizes boosted the IVC and SVC pressure drop but at the expense of lower arterial oxygen saturation and pulmonary flow. Excluding the nozzle size of 3.5 mm with the TCPC-state CO of 2.4 L/min, a positive correlation is observed between arterial oxygen saturation and the nozzle size. Higher AoG flow enhances the oxygen level in the AD flow but also causes more arterial desaturation as a result of increased AD flow, explaining the drop in oxygen levels in the aforementioned case (D_N_: 3.5 mm, CO_TCPC_: 2.4 L/min). Finally, CO exhibited a positive linear relationship with the aortic nozzle size, indicating higher ventricular load for larger nozzles. The ratio of CO over systemic flow was higher than the physiological threshold of 1.5 for aortic nozzle diameters of 3.0 mm and 3.5 mm, which is associated with a high risk of ventricular dysfunction and left ventricular volume overload. However, the ratio was strongly dependent on the TCPC-state CO as the aforementioned nozzle sizes did not exceed the physiological threshold in the case of TCPC-state CO of 3.5 L/min.

#### Device failure modes

**Figure 8:**
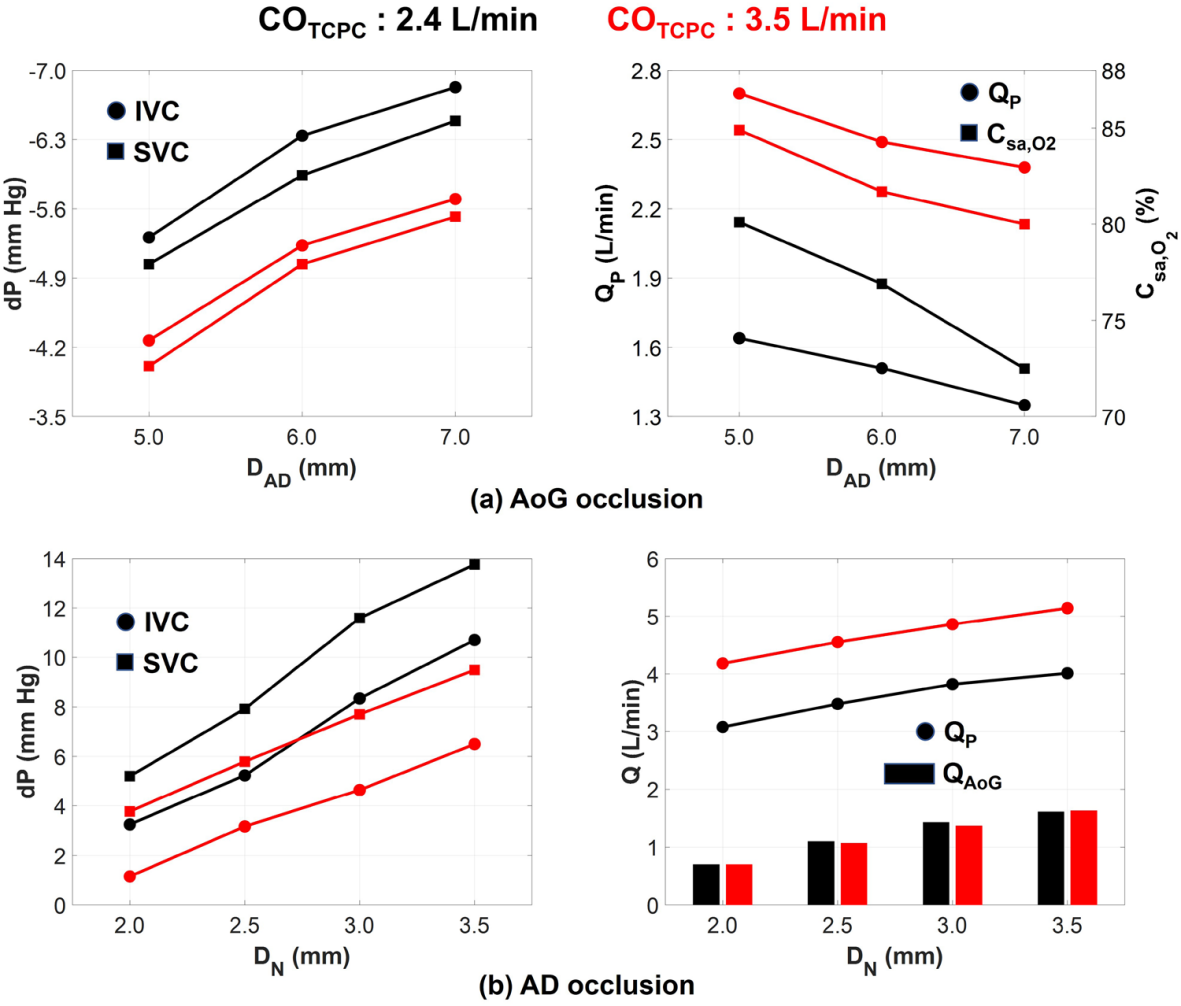
VEP-assisted Fontan hemodynamics in the event of device failure due to aortic graft (AoG) or atrial discharge (AD) occlusion. Occlusion of both grafts results in baseline Fontan hemodynamics. The ECC size and MAP are 13 mm and 85 mm Hg, respectively. *CO* cardiac output, *IVC* inferior vena cava, *SVC* superior vena cava, *dP* pressure change as compared to the TCPC-state condition, *Q*_*P*_ pulmonary flow, *C*_*sa,O2*_ arterial oxygen saturation, *D*_*AD*_ atrial discharge diameter, *D*_*N*_ aortic nozzle diameter, *Q*_*AoG*_ aortic graft flow, *ECC* extra-cardiac conduit, *MAP* mean aortic pressure, *VEP* venous ejector pump.

The summary of important Fontan hemodynamic indices in the event of VEP failure is illustrated in Fig. 8a,b for AoG and AD failure modes, respectively. The VEP was successful in maintaining low venous pressure levels but at the expense of significantly lower oxygen saturation during AoG failure mode. Atrial discharge diameters of 6 mm and 7 mm led to oxygen saturations significantly lower than 80% in the case of a TCPC-state CO of 2.4 L/min which is considered dangerously low. However, the oxygen saturation was strongly dependent on the CO as in the case of TCPC-state CO of 3.5 L/min, the aforementioned AD sizes maintained oxygen saturation levels higher than 80%. The occluded AoG failure mode exemplifies the conventional Fontan circulation with fenestrated hemodynamics. In the presence of AoG occlusion, the Fontan pressure registers lower compared to the full assist mode, owing to reduced pulmonary flow and a constant PVR. Conversely, in full assist mode, arterial oxygen saturation experiences a significant rise, albeit with a marginal reduction in IVC pressure drop. Ergo, the primary objective of the AoG graft is to maximize arterial oxygen saturation while minimizing the elevation in Fontan pressure due to increased pulmonary flow.

In the event of AD failure mode, venous pressure is the key hemodynamic index to monitor since no arterial oxygen desaturation exists. Considerable elevation in both IVC and SVC pressure levels was observed as compared to TCPC-state condition, which was positively correlated to aortic nozzle size and negatively correlated to TCPC-state CO. IVC pressure exhibited a lower elevation as compared to SVC due to the presence of jet Venturi effect. The pulmonary flow was significantly boosted as compared to both full-assist and TCPC-state conditions and the AoG flow was slightly lower than the full-assist mode. It is worth mentioning that PVR in these results was held constant and identical to its TCPC-state level. However, evidence of a negative correlation between PVR and pulmonary flow has been reported in the literature^57,58^. Although the quantitative data are limited, Schmitt et al.^57^ reported that a dobutamine-induced exercise condition with an increase in pulmonary flow was followed by a drop in PVR. The PVR percentage change was approximately in the range of 30–50%. To evaluate the impact of PVR drop in the event of AD failure mode, we assumed that the percentage drop in PVR is proportional to the percentage increase in pulmonary flow (as compared to TCPC-state condition) as long as it is lower than 50%. For higher pulmonary flow increase percentages, the PVR drop was assumed to stay at 50%. Table 2 summarizes the venous pressure data before and after the PVR change for the case with TCPC-state CO of 2.4 L/min. The IVC pressure exhibited a significant drop to levels lower than TCPC-state values for all nozzle sizes. The SVC, however, experienced a slight elevation in pressure which was varying based on aortic nozzle size. The SVC pressure rise was less than 0.5 mm Hg for the VEP with aortic nozzle sizes of 2 mm and 2.5 mm.

**Table 2:** Cycle-averaged venous pressure values in the event of atrial discharge graft occlusion and PVR drop obtained during pulsatile in vitro flow experiments.

D_N_ (mm)	PVR drop %	Vena cava	Pressure (mm Hg)		
			TCPC-state	VEP ADB	PVR drop
2.0	30	Inferior	15.8	19.05	14.13
		Superior	15.4	20.60	15.78
2.5	40	Inferior	15.8	21.03	13.24
		Superior	15.4	23.32	15.88
3.0	50	Inferior	15.8	24.14	12.78
		Superior	15.4	26.99	16.29
3.5	50	Inferior	15.8	26.50	13.73
		Superior	15.4	29.16	17.09

The results correspond to the TCPC-state cardiac output of 2.4 L/min. TCPC-state, VEP ADB, and PVR drop represent the baseline Fontan hemodynamics without VEP, the response of the system after atrial discharge graft occlusion, and the reduction of PVR values after occlusion, respectively.

*TCPC* total cavopulmonary connection, *PVR* pulmonary vascular resistance, *VEP* venous ejector pump.

### Impact of mean aortic pressure

**Figure 9:**
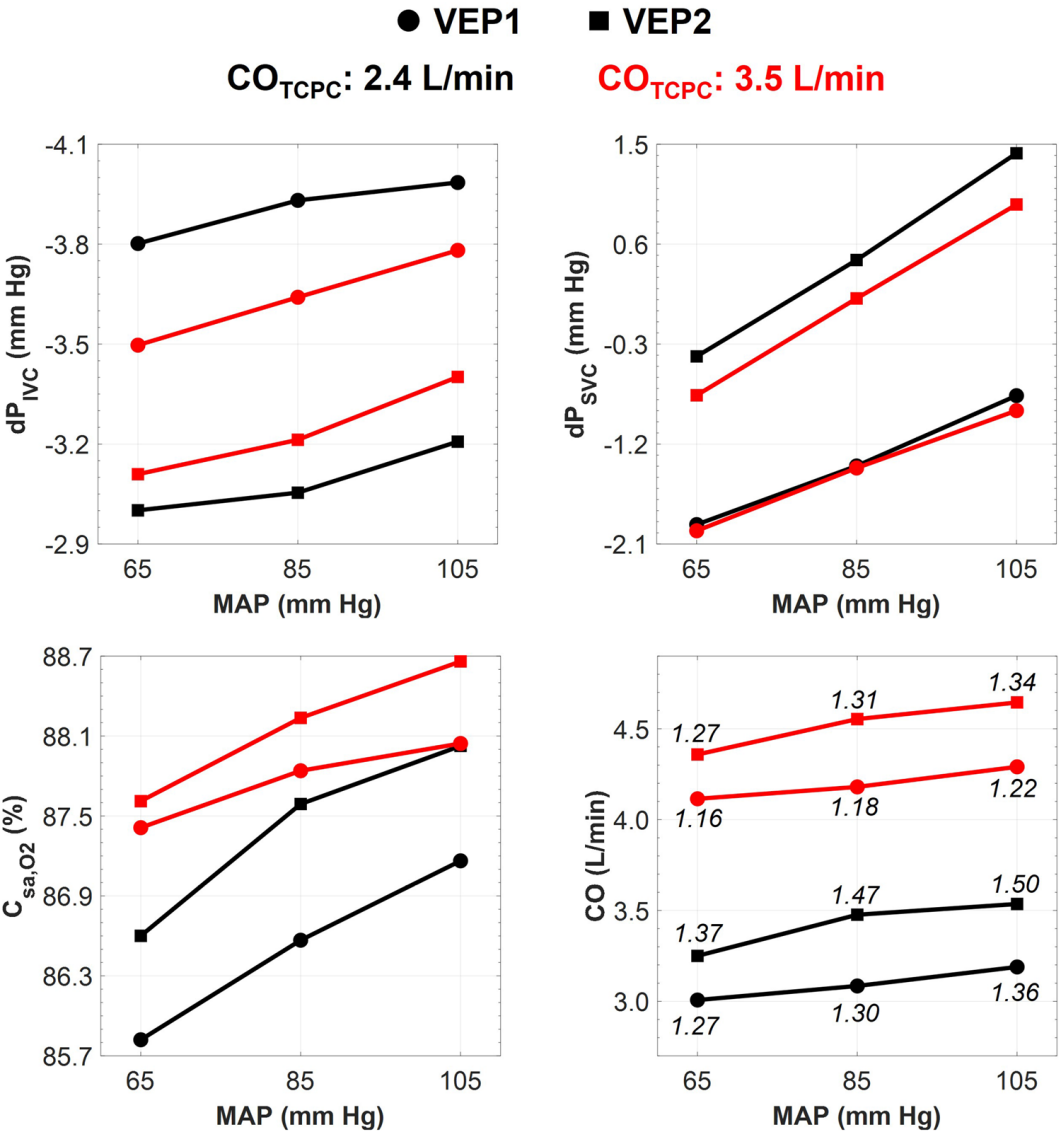
Impact of mean aortic pressure on the VEP-assisted Fontan hemodynamics during full assist mode. The ECC and atrial discharge sizes are 13 mm and 5 mm, respectively. VEP1 and VEP2 correspond to the designs with aortic nozzle sizes of 2.0 mm and 2.5 mm, respectively. *CO* cardiac output, *IVC* inferior vena cava, *SVC* superior vena cava, *dP* pressure change as compared to the TCPC-state condition, *C*_*sa,O2*_ arterial oxygen saturation, *ECC* extra-cardiac conduit, *MAP* mean aortic pressure, *VEP* venous ejector pump. TCPC-state represents the baseline Fontan hemodynamics without the VEP. The numbers on the CO chart represent the ratio of CO over systemic flow.

Two VEP prototypes with AD graft size of 5 mm and aortic nozzle sizes of 2.0 mm and 2.5 mm were selected and named VEP1 and VEP2, respectively. The selection was based on the hemodynamic indices presented in Section "Impact of geometrical parameters and cardiac output". AD graft sizes larger than 5 mm resulted in low arterial oxygen saturation levels (< 80%) in the event of AoG failure mode. More importantly, aortic nozzle diameters larger than 2.5 mm led to CO over systemic flow ratios higher than the physiological limit of 1.5, and thus, were not selected. The results in this section correspond to the EXP NO (II) in Table 1. Figure 9 summarizes the hemodynamic indices for various MAP. Mean aortic pressure was adjusted by tuning the SVR and keeping other physiological parameters unchanged. Higher MAP values improved performance by reducing IVC pressure and increasing oxygen saturation but with a slight elevation in SVC pressure. A change in MAP from 65 mm Hg to 105 mm Hg resulted in an average drop of 0.25 mm Hg in IVC pressure and an average increase of 1.45 mm Hg in SVC pressure over all cases. VEP1 maintained SVC pressure levels lower than those in the TCPC-state condition for all MAP values. Although cardiac output increased for higher MAP values, the ratio of cardiac output over systemic flow for both prototypes did not exceed the limit of 1.5 under any conditions.

### Impact of extra-cardiac conduit size and transpulmonary pressure gradient

**Figure 10:**
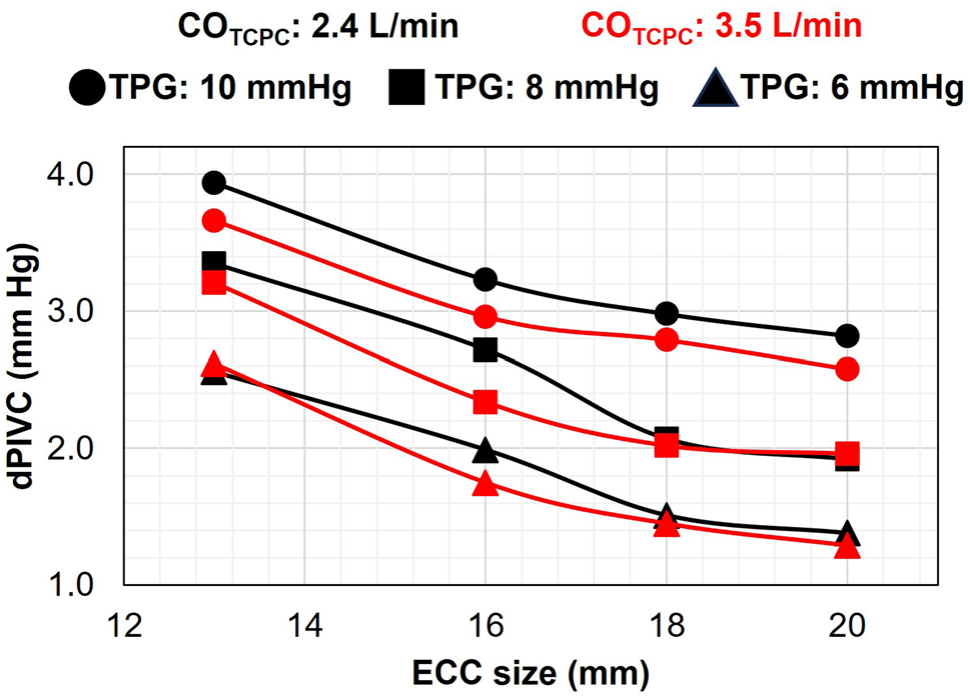
Impact of TPG and ECC size on the VEP (D_N_: 2.0 mm, D_AD_: 5.0 mm) performance at reducing IVC pressure during full assist in idealized TCPC. dP represents the change as compared to TCPC-state. *TPG* transpulmonary pressure gradient, *ECC* extra-cardiac conduit, *CO*_*TCPC*_ TCPC-state cardiac output, *TCPC* total cavopulmonary connection, *VEP* venous ejector pump, *D*_*N*_ aortic nozzle diameter, *D*_*AD*_ atrial discharge diameter, *IVC* inferior vena cava. TCPC-state represents the baseline Fontan hemodynamics without VEP.

Figure 10 presents a depiction of the VEP1 performance concerning diverse ECC sizes and TPG values in idealized TCPC models. The results in this section correspond to the EXP NO (III) in Table 1. An inverse relationship between ECC size and IVC pressure drop was evident. Specifically, an increase in ECC size from 13 to 20 mm resulted in reductions of 1.30 mm Hg and 1.35 mm Hg in IVC pressure drop for TCPC-state CO values of 2.4 L/min and 3.5 L/min, respectively, with a TPG of 10 mm Hg. This reduction in IVC pressure drop can be attributed to decreased jet momentum transfer in the presence of larger ECC sizes. In contrast, parameters such as cardiac output, pulmonary flow, and SVC pressure remained largely unaffected and exhibited minimal variations. Arterial oxygen saturation values were consistently maintained above 85% for all ECC sizes and TPG values; however, a slight decline was observed as the ECC size increased. Moreover, the TPG had a notable influence on VEP1 performance. Elevated TPG values led to increased IVC pressure drop and atrial discharge flow but concurrently resulted in lower arterial oxygen saturation levels and pulmonary flow. These findings highlight the intricate interplay of ECC size and TPG in shaping VEP performance within the TCPC environment.

### Impact of heart rate

**Figure 11:**
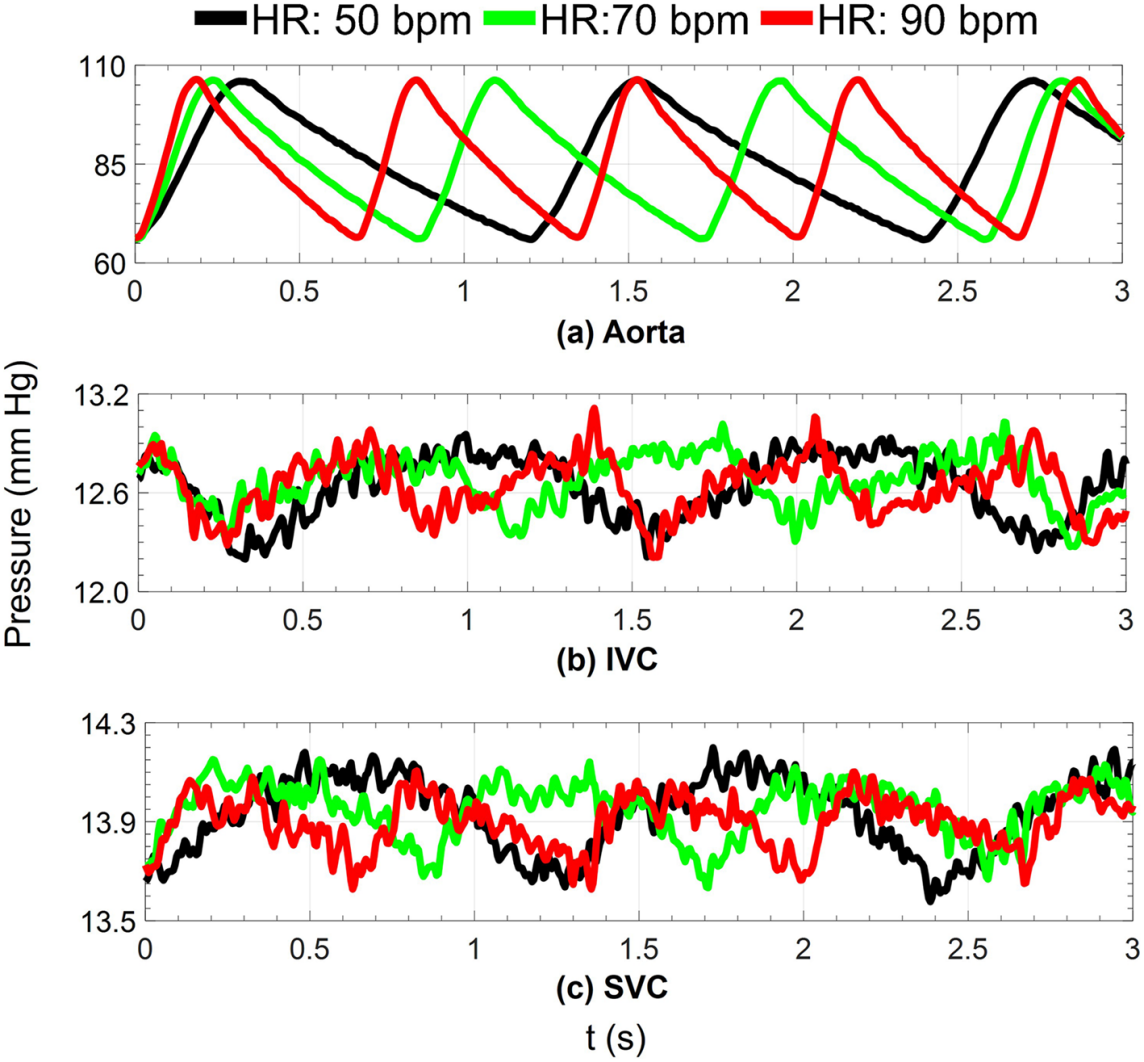
Impact of heart rate on aortic, IVC, and SVC instantaneous pressure waveforms during full assist operation of VEP with D_N_, D_AD_, and D_ECC_ of 2.0 mm, 5.0 mm, and 18 mm, respectively. *HR* heart rate, *IVC* inferior vena cava, *SVC* superior vena cava, *bpm* beat per minute, *D*_*N*_ aortic nozzle diameter, *D*_*AD*_ atrial discharge diameter, *D*_*ECC*_ extra-cardiac conduit size.

To evaluate the VEP performance under different heart rates, VEP1 with an ECC size of 18 mm was employed. Three heart rates of 50, 70, and 90 bpm were considered to encompass a broad range of potential variability. The results in this section correspond to the EXP NO (IV) in Table 1. Figure 11 depicts the instantaneous aortic and venous pressure waveforms during full-assist mode for each heart rate. Complementary to this, Table 3 provides a summary of cycle-averaged hemodynamic quantities. Observations revealed that higher heart rates corresponded to sharper venous pressure waveforms. While the heart rate influenced the instantaneous variations of venous pressure and other hemodynamic indices, the cycle-averaged quantities remained virtually unchanged. This underscores the insignificant impact of heart rate on VEP performance, indicating robust stability across varying heart rates.

**Table 3:** Impact of heart rate on the cycle-averaged VEP-assisted Fontan hemodynamics.

	CO_TCPC_: 2.4 L/min			CO_TCPC_: 3.5 L/min		
HR (bpm)	50	70	90	50	70	90
CO (L/min)	3.11	3.10	3.10	4.21	4.19	4.19
Q_s_ (L/min)	2.40	2.39	2.37	3.48	3.45	3.46
Q_P_ (L/min)	2.28	2.26	2.27	3.30	3.28	3.29
AoP (mm Hg)	85.01	84.89	85.02	84.66	85.08	84.96
IVCP (mm Hg)	12.67	12.69	12.64	13.11	13.08	13.16
SVCP (mm Hg)	13.94	13.96	13.91	14.27	14.21	14.27

The results correspond to the VEP1 with aortic nozzle, atrial discharge, and ECC size of 2.0 mm, 5.0 mm, and 18 mm, respectively.

*HR* heart rate, *CO* cardiac output, *Q*_*S*_ systemic flow rate, *Q*_*P*_ pulmonary flow rate, *AoP* aortic pressure, *IVCP* inferior vena cava pressure, *SVCP* superior vena cava pressure, *CO*_*TCPC*_ TCPC-state cardiac output, *bpm* beat per minute, *TCPC* total cavopulmonary connection, *VEP* venous ejector pump, *ECC* extra-cardiac conduit.

### Performance in the patient-specific model

Table 4 presents a summary of VEP1-assisted Fontan hemodynamics obtained using a patient-specific TCPC model. The results in this section correspond to the EXP NO (V) in Table 1. Notably, the performance observed in this patient-specific model closely resembles that of the idealized TCPC with an identical ECC size of 16 mm. This demonstrates the minor influence of TCPC complexity on VEP performance, highlighting the robustness and consistency of VEP performance across different TCPC configurations.

**Table 4:** Cycle-averaged VEP-assisted Fontan hemodynamic indices in patient-specific TCPC phantom.

	CO_TCPC_: 2.4 L/min		CO_TCPC_: 3.5 L/min	
	TCPC	TCPC + VEP	TCPC	TCPC + VEP
CO (L/min)	2.40	3.15	3.50	4.24
Q_S_ (L/min)	2.38	2.41	3.51	3.52
Q_P_ (L/min)	2.41	2.31	3.48	3.34
P_Ao_ (mm Hg)	85.29	84.90	85.22	85.34
P_IVC_ (mm Hg)	15.53	12.35	15.90	12.89
P_SVC_ (mm Hg)	15.35	13.80	15.69	14.37
CO / QS	1.00	1.31	1.00	1.20
dP_IVC_ (mm Hg)	–	− 3.18	–	− 3.01
dP_SVC_ (mm Hg)	–	− 1.55	–	− 1.32
C_sa,O2_ (%)	95	86.5	95.0	87.4

The patient-specific TCPC model has an ECC size of 16 mm. The results correspond to the VEP with an aortic nozzle and atrial discharge of 2.0 mm and 5.0 mm, respectively.

*CO* cardiac output, *Q*_*S*_ systemic flow rate, *Q*_*P*_ pulmonary flow rate, *P*_*Ao*_ aortic pressure, *P*_*IVC*_ IVC pressure, *P*_*SVC*_ SVC pressure, *dP* pressure change as compared to TCPC-state condition, *C*_*sa,O2*_ arterial oxygen saturation, *TCPC* total cavopulmonary connection, *VEP* venous ejector pump, *CO*_*TCPC*_ TCPC-state cardiac output. TCPC + VEP represents the full-assist mode. TCPC-state corresponds to the baseline Fontan hemodynamics without VEP.

## Discussion

The past 50 years have witnessed significant strides in the treatment of congenital heart disease, and single-ventricle palliation has emerged as a lifesaving development with immense implications. However, the pioneers of this technique foresaw potential limitations, recognizing the need for continuous refinement and modification to ensure its long-term viability with a normal lifespan^59–61^. Efforts aimed at reducing hemodynamic power loss through the implementation of optimized vascular connections have yielded insignificant outcomes, as they fail to address the inherent absence of a subpulmonic pump. In contrast, the utilization of externally powered circulatory systems as a means of cavopulmonary support to reinstate biventricular physiology is markedly restricted and accompanied by a notable risk of driveline infections and significant complications^62^. To alleviate the Fontan-associated disease due to elevated caval pressure, we earlier proposed a self-powered fully implantable solution with theoretical success in improving the local hemodynamics^43^. Unlike actively-powered systems, our solution provides passive cavopulmonary support which eliminates the ex vivo power requirement, leading to better patient mobilization and rehabilitation. This study aimed to validate the performance of the proposed solution and to assess its impact on the Fontan global hemodynamics by extensive in vitro experiments using an in-house pulsatile single-ventricle mock-up circulation loop.

The investigation demonstrated the profound reliance of the VEP on physiological states and geometric parameters. Nevertheless, it consistently provided IVC pressure reductions across all full assist scenarios, while ensuring arterial oxygen saturation levels were maintained above 80%. Activation of the aortic graft led to diminished arterial blood pressure, imposing a burden on the native ventricle due to augmented cardiac output. The extent of ventricular loading was markedly a function of the size of the aortic nozzle, displaying minimal susceptibility to variations in other physiological and geometrical factors. Notably, aortic nozzle sizes exceeding 2.5 mm can engender the development of ventricular dysfunction, atrial enlargement, and left ventricular volume overload, as evidenced by a CO to systemic flow ratio surpassing 1.5, a pivotal criterion for left-to-right shunts^63,64^, corroborating our theoretical findings^43^. Conversely, minor loads induced by smaller nozzles (≤ 2.5 mm) are tolerable to the native single-ventricle, given that Fontan failure primarily stems from diastolic dysfunction with preserved ejection fraction and ventricular systolic function in the majority of patients^65^. More importantly, the presence of atrial discharge improves preload, thereby enhancing myocardial performance and diastolic function. However, AD sizes exceeding 5 mm posed issues, as they led to suboptimal arterial oxygen saturation levels (< 80%) during instances of AoG occlusion, in accordance with our earlier computational predictions^43^.

The role of the aortic nozzle size in shaping the Fontan hemodynamics was investigated in-depth, and the results shed light on its pivotal impact. Larger nozzles were found to increase pulmonary flow and arterial oxygen saturation, yet this improvement came at the cost of reduced IVC pressure drop, increased ventricular load, and elevated SVC pressure. These observations were attributed primarily to the constant PVR and increased pulmonary flow, which contributed significantly to the higher caval pressure observed in cases with larger nozzles. Additionally, as the nozzle size increased, the inferior cavopulmonary pressure head rose, driven by the enhanced momentum transfer of the jet. The performance of the VEP was shown to be robust to variations in heart rate, as cycle-averaged indices remained virtually identical for heart rates ranging from 50 to 90 bpm. Moreover, a positive correlation was established between arterial blood pressure and VEP performance indices, including IVC pressure drop and arterial oxygen saturation. Regarding the influence of aortic pressure on VEP performance, although the cardiac output slightly increased with higher aortic pressures, the critical ratio of CO over systemic flow was consistently maintained below 1.5 for nozzle sizes smaller than 2.5 mm, even with aortic pressure varying from 65 to 105 mm Hg.

Notably, patient size significantly impacted IVC pressure drop, with the ECC size being a key determinant. An increase in the ECC size from 13 to 20 mm resulted in 1.23 ± 0.12 mm Hg (mean ± standard deviation) less IVC pressure drop due to reduced jet momentum transfer for larger sizes in VEP1. Moreover, the transpulmonary pressure gradient (TPG) exhibited a robust association with VEP-assisted Fontan hemodynamic indices. Reducing TPG from 10 mm Hg to 6 mm Hg led to a decrease of 1.30 ± 0.13 mm Hg and 1.43 ± 0.08 mm Hg in IVC and SVC pressure drop, respectively. Although higher TPG values were associated with lower arterial oxygen saturation levels, the VEP1 consistently maintained values above 85% under all conditions. Furthermore, it is noteworthy that a TPG of 6 mm Hg represents the threshold and early indicator of pulmonary hypertensive vascular disease in patients with cavopulmonary anastomosis, as per the European Pediatric Pulmonary Vascular Disease Network (EPPVDN)^53^. This emphasizes the VEP's capacity to provide support even in the initial stages of elevated venous pressure, with its efficacy improving as the condition progresses.

Prior studies investigating self-powered Fontan assist mechanisms have been limited in scope. An aortopulmonary shunt, designed to direct a portion of systemic flow into the pulmonary arteries, proved ineffective at reducing IVC pressure, owing to increased pulmonary flow and the constant PVR^40^. However, when the same shunt was positioned within the ECC lumen with fenestration, computational simulations reported a reduction in IVC pressure^41^. Despite this potential benefit, the proposed solution lacks clinical feasibility, as the unsupported shunt inside the ECC lumen is susceptible to pronounced oscillations due to highly pulsatile aortic flow and strong jet flow momentum. Moreover, the absence of failure scenario assessment and the presence of relatively large fenestration raise concerns about low arterial oxygen saturation levels in the event of shunt failure. In contrast, the VEP demonstrates the advantage of being thoroughly evaluated for fail-safe features under various failure scenarios, ensuring the maintenance of high arterial oxygen saturation levels (> 80%) and low IVC pressure in cases of AoG occlusion. In another approach, a shunt from the ascending aorta to the anastomosis of the SVC and pulmonary arteries was suggested to lower venous pressure^42^. While computational simulations revealed its potential for providing a modest venous pressure drop (~ 1 mm Hg), the authors assumed a constant pressure at the pulmonary arteries, which does not accurately reflect the hemodynamic response due to significantly increased pulmonary flow. More comprehensive multi-scale simulations or experimental studies would be necessary to fully assess its effectiveness. Additionally, the shunt resulted in highly unbalanced pulmonary flow, raising concerns about the potential development of pulmonary arteriovenous malformations.

Despite the in vitro outcomes indicating the favorable impact of the VEP on Fontan hemodynamics, the authors acknowledge that the solution remains conceptual. Further validation through in vivo animal trials is imperative, as in vitro circulatory loops lack the dynamic complexity inherent in human circulation. The potential for thromboembolism poses a primary concern in the in vivo implementation of VEP, given the presence of a high-velocity shunt with a small cross-section. Aggressive administration of pharmacologic anticoagulation may be necessary to mitigate the risk of thrombosis formation. Nonetheless, VEP exhibits the ability to sustain normal Fontan hemodynamics in the event of graft occlusion due to thrombosis, as fail-safe features were revealed in failure experiments. This study employed rigid TCPC phantoms. Although earlier research has suggested an insignificant impact of TCPC compliance on time-averaged Fontan hemodynamic indices^66^, the inclusion of an aortic graft significantly increases caval flow pulsatility, thereby rendering a compliant TCPC model a potentially more realistic representation. Additionally, the study did not investigate the influence of blood rheology on VEP performance; however, a blood analog closely matching the Fontan patients' blood viscosity was utilized. Moreover, the VEP's capacity to improve cavopulmonary pulsatility could potentially lead to enhanced endothelial function and lower PVR^36–38^, positively influencing VEP performance. These important factors, while not addressed in this study, may further enhance the VEP performance and should be explored in future investigations.

It is worth noting that the efficacy of the proposed therapy is contingent on a functional ventricle with preserved ejection fraction. Patients with impaired myocardial contractility and systolic dysfunction would not benefit from this solution. The ideal timing for implementing VEP, whether during the initial Fontan operation or later, is uncertain and beyond the scope of this study. Nevertheless, the authors believe that Fontan patients with healthy caval pressure do not require the proposed therapy and the VEP implantation can be considered after diagnosing elevated Fontan pressure, especially in conjunction with the onset of protein-losing enteropathy. Moreover, a 13 mm ECC size was utilized to identify the optimal VEP geometrical parameters. Although we acknowledge the current clinical preference for larger ECC sizes, the validity of identified sizes is maintained as the IVC pressure was the only quantity exhibiting significant sensitivity to ECC size with other hemodynamic indices minimally affected. The last but not the least, The safety of VEP during AD occlusion was relied on the assumption of reduced PVR with increased pulmonary flow^57^. Nevertheless, the cited study has notable limitations, with a particularly small sample size of 10 patients being a significant concern. Moreover, the identification of 2 out of the 10 patients as non-responders due to minimal changes in PVR following dobutamine administration adds a critical dimension to the limitation of our assumption. The authors acknowledge the limitations associated with the underlying assumption and speculate that in case of impairment of pulmonary vascular reserve, percutaneous septal occlusion techniques may offer a viable approach for closing the aortic graft during AD occlusion, consequently achieving a TCPC-state (baseline Fontan) circulation. Finally, the heart rate was assumed to remain identical pre and post VEP implementation, attributing the increase in cardiac output to an increase in stroke volume. While the authors recognize the increased heart rate in response to decreased systemic pressure, our investigation, detailed in Section "Impact of heart rate", revealed that the influence of heart rate on cycle-averaged hemodynamic quantities was inconsequential. Thus, the assumption of a constant heart rate is deemed valid based on our findings.

## Conclusion

The effectiveness of a passive solution addressing elevated IVC pressure in Fontan patients was rigorously validated through extensive in vitro experiments. The proposed solution represents a fully implantable, intracorporeal approach, characterized by its simplicity in structure and clinical feasibility. A multi-scale pulsatile in vitro mock-up circulatory loop was developed to emulate Fontan circulation, facilitating the assessment of VEP performance under various physiological and geometrical conditions simulating post-Fontan pediatric stages. Notably, the VEP demonstrated success in reducing IVC pressure while consistently maintaining high arterial oxygen saturation levels. These results highlight the potential of the VEP as a possiblesolution for managing elevated IVC pressure in Fontan patients.

### Supplementary Information

Supplementary Information.

## Data Availability

All data generated or analyzed during this study are included in this published article and the supplementary material.
