# Supplementary material for: In vitro hemodynamic performance of a blood pump for self-powered venous assist in univentricular hearts
Source: Sci Rep. 2024 Mar 23;14:6941. doi: 10.1038/s41598-024-57269-7 (PMC10960831; doi:10.1038/s41598-024-57269-7)
Supplement: Supplementary file 1 — Supplementary Information. [file 41598_2024_57269_MOESM1_ESM.pdf]

Supplementary Material

In Vitro Hemodynamic Performance of a Blood Pump for Self-powered Venous Assist in Univentricular Hearts

Reza Rasooli1, Henrik Holmstrom, Knut Erik Teigen Giljarhus , Ingunn Westvik Jolma, Jan Ludvig Vinningland, Charlotte de Lange, Henrik Brun, Aksel Hiorth

1. Post-VEP cardiac response assumptions and justifications

The introduction of the Venous Ejector Pump (VEP) into the circulation (Full assist mode) leads to decreased systemic pressure due to the presence of a left-to-right shunt which directs a portion of the systemic flow to the VEP. The level of systemic pressure drop is significantly dependent on the size of the aortic graft size, as reported in the Section 3.1.1 of the article main text. In this case, the baroreceptor reflex plays a crucial role in regulating systemic blood pressure and attempting to maintain it within a relatively constant range. Increased sympathetic activity due to baroreceptor activation leads to vasoconstriction of blood vessels, including the arteries, resulting in increased systemic vascular resistance (SVR). The sympathetic activation also stimulates the heart, leading to an increase in heart rate and stroke volume. This increased cardiac output (CO) helps compensate for the decreased systemic pressure by enhancing blood flow into the systemic circulation. In this study, we assumed that the regulation of the decreased systemic blood pressure is achieved through adjustments in CO. While the authors acknowledge that baroreceptor activation, prompted by decreased systemic pressure, is linked to increased CO and SVR, any compensatory impact from SVR in response to the reduced systemic pressure yields a relatively lower increase in CO. Consequently, this leads to a higher IVC pressure drop, contributing to better performance. The underlying mechanism can be attributed to the lower increase in pulmonary flow, resulting in a proportional decrease in venous pressure elevation due to the constant PVR. Therefore, systemic pressure regulation through pure CO adjustment reveals the pump’s poorest performance, a critical consideration to prevent overestimation. In order to validate this hypothesis

empirically, we conducted experiments by regulating systemic pressure through SVR adjustments. The dataset presented in Table 1 delineates key cycle-averaged hemodynamic parameters, comparing scenarios of systemic pressure regulation through SVR versus CO. Significantly, the SVR-regulated scenario exhibits a noteworthy reduction in venous pressure levels compared to the CO-regulated scenario, conclusively delineating the latter as indicative of the pump's least efficient performance.

Table 1. Cycle-averaged hemodynamic indices during both TCPC-state and full assist mode with different systemic pressure regulation mechanisms. TCPC-state: baseline Fontan circulation without the VEP, TCPC+VEP: Fontan circulation with the pump operating, Acute response: the immediate hemodynamic response after the VEP introduction, SVR-regulated: regulation of decreased systemic pressure though SVR, CO-regulated: regulation of decreased systemic pressure through CO, CO: cardiac output, Q_S: systemic flow rate, Q_P: pulmonary flow rate, Q_{AoG}: aortic graft flow rate, Q_{AD}: atrial discharge graft flow rate, P_{Ao}: aortic pressure, P_{IVC}: inferior vena cava pressure, P_{SVC}: superior vena cava pressure, dP: pressure change as compared to TCPC-state baseline condition, $C_{sa,O2}$: systemic arterial oxygen saturation.

	**TCPC-state**	**TCPC+VEP**		
	**Baseline**	**Full Assist Acute Response**	**Full Assist SVR-regulated**	**Full Assist CO-regulated**
**CO (L/min)**	3.50	3.50	3.50	4.55
**Q_S (L/min)**	3.50	2.76	2.36	3.48
**Q_P (L/min)**	3.49	2.68	2.70	3.63
**Q_{AoG} (L/min)**	0.00	0.74	1.14	1.07
**Q_{AD} (L/min)**	0.00	0.82	0.80	0.93
**P_{Ao} (mm Hg)**	85.1	57.6	85.1	84.9
**P_{IVC} (mm Hg)**	16.2	10.7	10.1	13.0
**P_{SVC} (mm Hg)**	15.6	12.4	12.1	15.7
**dP_{IVC} (mm Hg)**	------	-5.5	-6.1	-3.2
**dP_{SVC} (mm Hg)**	------	-3.2	-3.5	0.1
**$C_{sa,O2}$ (%)**	95	88	88	88

More importantly, there is an absence of a universally recognized quantitative framework elucidating the compensatory roles played by both CO and SVR in response to a significant drop in systemic pressure. In essence, our current understanding, to the best of our knowledge, does not offer a quantitative understanding of the proportion by which decreased systemic pressure is compensated for by changes in cardiac output as opposed to adjustments in SVR.

In this study, the authors also assumed that the heart rate remains constant, attributing the increase in cardiac output to an increase in stroke volume. While we recognize that the heart rate may indeed elevate in response to decreased systemic pressure, our investigation, detailed in Section 3.4 of the article main text, revealed that the influence of heart rate on cycle-averaged hemodynamic quantities was inconsequential. Thus, the assumption of a constant heart rate is deemed valid based on our findings. Moreover, the assumption that arterial pressure remains in a relatively constant range long term after the VEP is introduced to the circulation is an ideal simplification made in our study. In reality, the effect of the aortic graft on arterial pressure can depend on various factors, including the magnitude of the shunt, the responsiveness of the cardiovascular system, and compensatory mechanisms. In some cases, the compensatory mechanisms may effectively maintain arterial pressure, resulting in a relatively constant level. However, the extent to which arterial pressure remains constant can vary among individuals and may be influenced by factors such as overall cardiovascular health, the presence of other medical conditions, and the specific characteristics of the shunt. While the authors acknowledge the intricacies inherent in cardiovascular dynamics, we believe that, within the scope of our study, assuming a relatively stable systemic pressure is a reasonable assumption. This choice is underpinned by the modest size of the shunt, featuring a cardiac output to systemic flow ratio of less than 1.5, prior in vitro studies in the literature [1, 2] and more importantly, the minor impact of aortic pressure variation on device's performance discussed in Section 3.2 of the article main text.

REFERENCES

1. Das, A., et al., *In-vitro validation of self-powered fontan circulation for treatment of single ventricle anomaly.* Fluids, 2021. **6**(11): p. 401.
2. Pekkan, K., et al., *In vitro validation of a self-driving aortic-turbine venous-assist device for Fontan patients.* The Journal of thoracic and cardiovascular surgery, 2018. **156**(1): p. 292-301. e7.
